# Social Participation, Social Environment and Death Ideations in Later Life

**DOI:** 10.1371/journal.pone.0046723

**Published:** 2012-10-08

**Authors:** Thomas Saïas, François Beck, Julie Bodard, Romain Guignard, Enguerrand du Roscoät

**Affiliations:** 1 Direction des affaires scientifiques, Institut National de Prévention et d’Education pour la Santé, Saint-Denis, France; 2 Département de psychologie, Université du Québec à Montréal, Montréal, Canada; 3 Cermes3 - Cesames team, CNRS UMR 8211, Inserm U988, Université Paris Descartes, Sorbonne Paris Cité, EHESS, Paris, France; Cardiff University, United Kingdom

## Abstract

**Objective:**

Few studies on elders’ suicide and depression have integrated social and community factors in their explicative models. Most of the studied variables used are focused on individual and based on psychopathological models. The purpose of this study is to investigate the impact of socio-environmental factors on death ideations, using data from the European SHARE cohort.

**Method:**

Social support components and death ideations have been studied, together with known individual risk factors, within a sample of 11,425 European participants in the SHARE study, aged over 64. The item evaluating death ideations was extracted from the EURO-D12 questionnaire.

**Results:**

The high prevalence of death ideations (6.9% for men and 13.0% for women) confirmed that elders’ death ideations, as it is known to be linked to suicidal behaviors, is a major public health issue. Bivariate analyses revealed a strong association between community participation and death ideations. This association was no longer significant while adjusting for depressive symptomatology. The logistic model identified that factors significantly associated with death ideations, when adjusted for the other factors were: having multiple depressive symptoms (OR = 1.64 per symptom) being aged, especially over 84 (OR = 1.58), being retired for fewer than five years (OR = 1.46), being widowed (OR = 1.35) and having a long-term illness (OR = 1.28).

**Conclusions:**

Although social and community participation is associated to death ideations, this link becomes non-significant in a regression model taking into account other factors. It is important to notice that depressive symptoms, which are obviously closely related to death ideations, take the greatest part in the association among all associated factors. Our results suggest that, consistently with the literature, while addressing death ideation or suicide prevention, professionals have to consider first the secondary prevention of depressive symptomatology. Strategies targeting social isolation and community participation should be considered as part of primary prevention policies.

## Introduction

The field of research in elders suicide (in this paper, we will focus on people over 65) and its prevention faces two opposing dynamics. Whereas most of the published articles since 1990 have concentrated on identifying suicide risk factors and have recommended developing preventive strategies targeting these factors, only a few studies have looked at preventive actions themselves.

Moreover, regarding research projects on suicide risk factors, only a few have focused on social and community factors, or integrative models, with most of these studies used individual and psychopathological models. We have criticized this specific focus on individual determinants of suicide or suicidal thoughts in older people [1], especially for the impact of depression [2] and somatic disorders [3], [4]. Hence, in a recent review, Lapierre et al. [5] mentioned this phenomenon, referring to “suicidal individuals”, with no parallel reference to suicidal environment or context.

Therefore, this research field is both highly investigated (with the interest in individual risk factors) and ignored (regarding studies focusing on the social determinants of suicide, suicidal thoughts or death ideations, and studies on preventive actions of suicide in seniors).

In 2002, the World Health Organization (WHO) published alarming numbers related to the incidence of senior suicide with an average rate of 30/100,000 [6], ranging from 5.7/100,000 in Greece to 52.7/100,000 in Switzerland in people over 75 [7]. In France, it has been shown that suicide rates in those 75–84 years old were three time higher than those in 25–34 years old, although it remains a secondary cause of death (0.7%) after 65 [8]. Therefore, since suicide in elders remains a marginal problem, in comparison to other health issues in seniors -and given that it constitutes one of the leading cause of death in young (15–34) people -, older people, as verified in different national contexts, are not considered as a population with specific priority prevention needs [9].

Although difficult to estimate accurately (in particular because of *passive suicides,* most of which are due to stopping medication prescribed for a chronic disease), rates of suicides in elders are high. In Europe, it has been shown that up to 20% of older people declared having death thoughts or wishes (e.g. [10], [11]). This calls for researchers to develop specific preventive actions targeting suicide determinants, such as death ideations, depression, access to harmful medication or lethal means [12], and the factors of these determinants (social isolation, community participation, social attitudes toward and representations of older persons, etc.).

The field of research in suicide ideation and death ideations has been growing since the last 20 years. It has been shown that death ideations were associated with risk factors such as poor health conditions (disability or poor perceived health -e.g. [13]-, sensory impairment -e.g. [14]-), being widowed or single [13] or low perceived social support [13].

In a recent review, Rurup et al. [15] investigated the effects of social network and loneliness on death ideations. Social network was evaluated through the number of adults that were regularly in contact with the participants, and whom were perceived as important for them. Loneliness was assessed with a cognitive theoretical approach assuming that high discrepancy between expected support (affection and intimacy) and actual received support was characteristic of a subjective feeling of loneliness.

The authors pointed out that, even when controlling for depression, small social networks (OR = 1.1; CI 95% = 1.0–1.1, p = .03) and loneliness (OR = 2.3; CI 95% = 1.1–4.8, p = .03) were positively associated with death ideations (current wish to die).

In a another review by Van Orden and Conwell, based on the interpersonal theory of suicide [16], social disconnectedness was pointed out, together with depression, somatic diseases and functional impairment, as a major risk factor for suicide [17]. Based on these data, the authors have recommended as a primary preventive strategy to increase connectedness and to reinforce psychotherapy based on interpersonal and social issues in elders with high risk of suicide.

In another case-control study by Turvey et al. on 21 older people that committed suicide, amongst the EPESE cohort (14,500 older people in the United States), the authors have stated in their bivariate analyses that people with a low emotional network (close friends and family participants declared being closed to) were more likely to commit suicide [18]. Though, these associations were not tested in multivariate models controlling for depression.

This also applies to the impact of social interactions on wider mental health conditions. In the Boen, Dalgard and Bjertness [19] study of the associations between psychological distress and social support, the authors have reported that the number of close friends was negatively associated with psychological distress (OR = 0.61; 95% CI = 0.47–0.80). They also reported that lack of social support was directly related to psychological distress, while adjusting for socio-demographical variables and poor health conditions.

This interest in social isolation as a risk factor for suicide is growing, as epidemiological data underline the high number of isolated elders. In the Shankar, McMunn, Banks and Steptoe English study of 8,688 people (ELSA cohort), 7% of the participants were extremely isolated (isolation was assessed with a five-item instrument evaluating marital status, the monthly number of contact with participant’s children, number of contacts with other relatives or other friends, and community participation) [20]. Nevertheless, few studies have looked at social isolation as a target for preventive actions in elders. Cognitive-behavioral approaches to promote adaptation to retirement [21], online emotional support aiming at promoting perceived social support [22], [23], or interpersonal psychotherapy [24] are the most common approaches related to interpersonal support and social isolation that can be identified [5]. Using an ecological perspective [25], one can say that further studies should shift from an interest in individual determinants to a focus on ecological determinants, i.e., on social, territorial and societal health determinants. This approach is in line with Durkheim’s initial conclusions on the importance of social inclusion for an optimal physical and mental health [26].

**Table 1:** Sample description and bivariate analyses (n = 11,440).

	N	weighted %	Death ideation (weighted%)	F	P
Gender					
Male	5,227	41.1	6.9		
Female	6,213	58.9	13.0	39.74	<.001
Age					
65–69	3,648	30.2	7.1		
70–74	2,976	24.2	8.0		
75–79	2,437	21.9	11.0		
80–84	1,489	14.5	15.9		
Over 84	890	9.1	19.5	17.09	<.001
Country					
Austria	736	*	6.7		
Germany	936		7.9		
Belgium	1,085		9.8		
Denmark	594		5.5		
Spain	825		11.6		
France	1,097		13.2		
Greece	1,134		6.5		
Netherlands	873		6.6		
Italy	1,168		11.2		
Poland	792		16.9		
Czech Republic	746		11.6		
Sweden	815		5.8		
Switzerland	629		7.6	6.58	<.001
Education					
Less than 12 years	8,344	74.7	11.5		
12 years and over	3,014	25.3	7.4	13.62	<.001
Marital Status					
Married/registered partnership	7,262	56.7	7.6		
Divorced/Separated	584	4.9	9.3		
Single	479	5.3	10.0		
Widowed	3,033	33.1	15.8	19.51	<.001
Income perception					
Insufficient	4,596	42.7	13.6		
Sufficient	6,844	57.3	8.2	31.69	<.001
Tobacco use					
No	10,004	90.0	10.4		
Yes	1,260	10.0	10.9	0.08	.76
Chronic alcohol abuse					
No	10,656	95.3	10.7		
Yes	573	4.7	5.7	5.43	.02
Long term illness					
No	5,088	39.9	6.6		
Yes	6,317	60.1	13.1	44.38	<.001
Retired less than 5 years					
No	9,653	90.6	10.6		
Yes	1,189	9.4	6.6	7.33	.006
Type of neighborhood					
Big city	1,477	14.4	9.6		
Surburbs or outskirts of a big city	1,666	12.0	13.4		
Large town	2,152	15.3	10.5		
Small town	2,864	27.1	9.9		
Rural area or village	3,281	31.3	10.3	1.34	.24
Children					
No	1,176	11.4	12.5		
Yes	10,264	88.6	10.2	2.03	.15
Cohabitation with children					
No	7,699	70.7	10.0		
Yes	2,549	29.3	10.7	0.34	.56
Contacts with children outside participant’s residence					
No	836	12.8	12.2		
Yes	6,860	87.2	9.7	1.46	.23
Recieved material support in the last 12 months					
No	8,399	72.0	8.9		
Yes	3,035	28.0	14.8	28.5	<.001
Gave material support in the last 12 months					
No	8,949	80.9	11.4		
Yes	2,414	19.1	6.7	16.76	<.001
Participated in community activities in the last month					
No	6,674	65.3	12.4		
Yes	4,616	34.7	6.7	33.87	<.001
Death ideation in the last month					
No	10,239	89.5			
Yes	956	10.5			
Mean number of positive items at Euro-D (11 items) for participants with and without death ideations					
Positive death ideations item	5.56				
Negative death ideations item	2.43				

* Weighted percentages by country are not showed since data were not adjusted for country size.

The purpose of this paper is to contribute to investigate the impact of socio-environmental factors on death ideations in older people, using data from the European SHARE cohort [27]. Social support variables components and death ideations have been studied within a sample of 11,425 European participants aged over 65. We have hypothesized that, in addition to the individual risk factors of death ideations (gender, age, recent retirement, widowhood, functional impairment, depressive symptoms, etc.) that have been identified in the literature, an effect of the different dimensions of social support (material support, social inclusion, community participation) could be observed.

**Figure 1:**
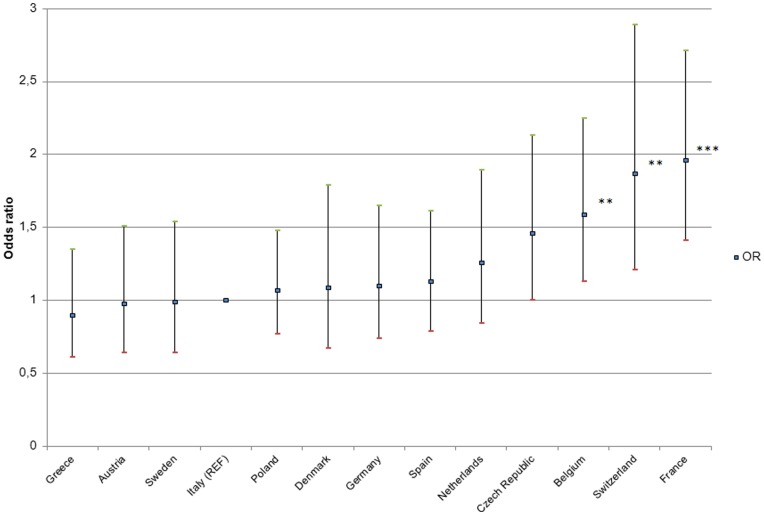
Death ideation by country. (Adjusted odds ratio, Italy as reference, ***P<.001, **P<.01.).

## Materials and Methods

This research used data from the European SHARE (*Study of Health, Ageing and Retirement in Europe*) cohort [27]. SHARE is a longitudinal study on over 45,000 participants from 15 different European countries (Denmark, Sweden, Belgium, Poland, Czech Republic, The Netherlands, Germany, Austria, Switzerland, France, Spain, Italy, Ireland, Israel and Greece) and aimed at evaluating the health, social and economic conditions of people over 50 in Europe. Data from the latest SHARE wave (second wave, 2006–2007) were used for this study. This face to face survey evaluated 34,415 people in all countries listed earlier except Israel. Participation rate was 72%. Sampling weights for Ireland were not available at the time of our study, and we decided to exclude this sub-sample. Participants over 65 were selected to be included in the analyses, resulting in a final sample of 11,425 participants who answered all the necessary items to perform the sample weighting.

The item evaluating death ideations (“In the last month, have you felt that you would rather be dead?”) was extracted from the EURO-D12 questionnaire [28], a specific trans-European questionnaire evaluating depression, derived from the *Geriatric Mental State Examination* [29].

**Table 2:** Logistic model predicting death ideation.

	OR	p	IC 95%	Z
Gender				
Male	1.0			
Female	1.15	.119	[.96–1.37]	1.56
Age				
65–69	1.0			
70–74	1.21	.106	[.96–1.53]	1.62
75–79	1.38	.01	[1.08–1.76]	2.58
80–84	1.34	.04	[1.02–1.77]	2.08
Over 84	1.59	.005	[1.15–2.18]	2.82
Education				
Less than 12 years	.94	.514	[.77–1.14]	−0.65
12 years and over	1.0			
Marital Status				
Married/Registered partnership	1.0			
Divorced/Separated	1.19	.336	[.83–1.71]	0.96
Single	1.19	.390	[.80–1.76]	0.86
Widowed	1.35	.002	[1.12–1.63]	3.09
Income perception				
Insufficient	1.08	.422	[.90–1.28]	0.80
Sufficient	1.0			
Alcohol abuse				
No	1.0			
Yes	.81	.343	[.52–1.25]	−0.95
Long-term illness				
No	1.0			
Yes	1.28	.008	[1.07–1.53]	2.67
Retired less than 5 years				
No	1.0			
Yes	1.46	.015	[1.07–1.98]	2.42
Recieved material support in the last 12 months				
No	1.0			
Yes	1.12	.197	[.94–1.33]	1.29
Gave material support in the last 12 months				
No	1.0			
Yes	.96	.724	[.77–1.20]	−0.35
Participated in community activitiesin the last month				
No	1.0			
Yes	.90	.259	[.75–1.08]	−1.13
Score Euro-D (11 items)				
Per each positive item	1.64	<.001	[1.59–1.70]	27.61

Hosmer-Lemeshow chi2(8) = 21.88; p<.05.

Pseudo R^2^ = 0.23.

The model is adjusted for country.

The other 11 items from the EURO-D12 were computed in a continuous score (number of depressive symptoms) as a proxy for evaluating depression.

Material support (received/given) was evaluated using a material social support item received or given in the last 12 months. Type of support included (a) personal help i.e., dressing, including putting on shoes and socks, bathing or showering, eating, getting in or out of bed, using the toilet, including getting up or down, etc.; (b) practical household help, e.g., with home repairs, gardening, transportation, shopping, household chores, etc.; and (c) help with paperwork, such as filling out forms, settling financial or legal matters.

Community participation in the last month was assessed through the participants’ declaration of taking part in at least one activity such as voluntary or charity work, caring for a sick or disabled adult, providing help to family, friends or neighbors, attending an educational or training course, going to a sport, social or other kind of club, taking part in a religious organization, taking part in a political or community-related organization.

Socio-demographical data (age, country, gender, education, length of retirement, marital status, income) were analyzed, as well as health data (long-term illness, use of tobacco, chronic alcohol abuse) known to be associated with increased suicidal risk [12], [30], [31], and socio-ecological data, which were the specific object of this study: type of neighborhood, number of children and frequency of contacts with children, intergenerational cohabitation, material support received, material support given, and community participation.

Missing data were imputed for income perception, number of children and type of neighborhood.

Poverty was assessed using an item of subjective perception (“Thinking of your household’s total monthly income, would you say that your household is able to make ends meet?”) with answers “with great difficulties” and “with some difficulties” identified as a measurement of poverty in order to have a common measurement in the 13 participating countries, with various minimum incomes for retired people.

Chronic alcohol abuse was assessed using a threshold of 14 alcohol units per week in women and 21 units in men.

Long-term illness was assessed using the answer to the item “Do you have any long-term health problems, illness, disability or infirmity?”.

Data were analyzed using STATA® v.10.1. Sample descriptions using all variables and bivariate analyses using social, socio-demographical, health and death ideations data were drawn up. Then, a logistic model including all significant variables from the bivariate analyses (Pearson’s chi-square with Rao-Scott second order correction -Fisher’s value-) was performed. All data were entered simultaneously. Data were weighted in univariate and bivariate analyses, taking into account the probability of interviewee selection within the household and the national population structure in terms of gender, age and geographic area.

## Results

Table 1 displays the final sample description (n = 11,440) together with bivariate analyses between social, health and socio-demographical data and the death ideations item.

Death ideation was present in 6.9% (men) and 13.0% (women) of the SHARE sample.

From the participants who have declared death ideations, the mean number of depressive symptoms as assessed with the 11 other items of the Euro-D12 was 5.56, compared with 2.43 in participants who did not declared death ideations.

Age (F [3.8, 42,542.8] = 17.09; *P<*.001), marital status (F [2.9, 33,113.4] = 19.51; *P<*.001) and country (F [6.0, 67,556.6] = 6.58; *P<*.001) displayed a strong association with the dependent variable.

Women (F [1, 11,170] = 39.74; *P<*.001), participants with fewer than 12 years of education (F [1, 11,116] = 13.62; *P<*.001), participants receiving insufficient income (F [1, 11,170] = 31.69; *P<*.001) or having a long-term illness (F [1, 11,166] = 44.38; *P<*.001) were more at-risk for displaying death ideations.

Giving material support (F [1, 11,143] = 16.76; *P<*.001) or participating to community activities (F [1, 11,119] = 33.87; *P<*.001) were negatively associated to death ideations, whereas receiving material support was positively associated to such ideations (F [1, 11,165] = 28.54; *P<*.001).

Chronic alcohol abuse was negatively related to suicidal thoughts (F [1, 11,016] = 5.43; *P = *.02). Lastly, without controlling for age, being retired for fewer than five years was negatively related to death ideations (F [1, 10,660] = 7.33; *P = *.006).

However, tobacco use, type of neighborhood, having children, cohabitating with children or being in contact with children were not associated with death ideations in the last 12 months.

Figure 1 presents the results observed in each country. Differences were identified. Italy was entered as the reference country, because of its largest sample. Regression analyses showed that French participants were the most vulnerable to death ideations (OR = 1.96, IC 95% = 1.41 to 2.71; *P*<.001), while Greeks (OR = .90, CI 95% = .61 to 1.35; *ns*) had the lower levels of suicidal thoughts.

Regression analyses partially confirmed bivariate associations, and invalidate the positive association between death ideations and gender, income perception, low level of education, alcohol abuse, social support (both given and received) and social participation while controlling for the other factors in the model.

Score to the 11 other items of the Euro-D (proxy for depression) was highly related to death ideations, with an odds ratio for each positive item of 1.64 (CI 95% = 1.59 to 1.70; *P<*.001).

The logistic model (Table 2) shows that factors associated with death ideations were: widowing (OR = 1.35, CI 95% = 1.12 to 1.63; *P = *.002), having a long-term illness (OR = 1.28, CI 95% = 1.07 to 1.53; *P = *.008), being aged, especially over 84 (OR = 1.58, CI 95% = 1.15 to 2.18; *P = *.005),, being retired for fewer than five years (OR = 1.46, CI 95% = 1.07 to 1.98; *P = *.015).

## Discussion

This research aimed to identify the respective weight of risk factors of death ideations among people aged more than 65 years, using data from the SHARE European study. According to the lack of evidence in this field, a particular glance was addressed on the social and interpersonal risk factors.

Death ideations rates were high (6.9% in men, 13.0% in women) in the European SHARE sample. We found a strong variability in death ideations in the countries participating in the SHARE survey. These results are consistent with those of Kelly, Davoren, Mhaolain, Breen and Casey [32], who pointed out an association between the social trust, i.e., the confidence expressed by citizens between each other, and the rates of suicide in 11 European countries. In a study using data from 26 European countries, Yur’yev et al. [33] also found a negative association between the societal representations of people aged more than 70 years and the rate of suicide in the elderly. If the results by country tally only partially with those of our research, they nevertheless lead us to consider suicide risk factors such as death ideations as multifactorial, integrating a major social and societal component.

Results from the multivariate analyses partially confirmed data from the literature. The depressive symptomatology (OR = 1.64 per each positive item from the 11-item Euro-D12, CI 95% = 1.59–1.70; *P<.*001) was strongly related to death ideations, confirming what is known about the link between depression and death or suicidal thinking (e.g [5], [34]). Other known individual risk factors were associated with death ideations in our model, even while controlling for the high effect of depression. Hence, being retired from less than 5 years (OR = 1.46, CI 95% = 1.07 to 1.98; *P = *.015) was confirmed as an important risk factor for developing death ideation.

The effect of age was also pointed out, in accordance with most of the previous researches on depression and suicide in elders. Hence, being aged, more specifically over 84 was retrieved as a major risk factor (OR = 1.58, CI 95% = 1.15 to 2.18; *P = *.005) for developing death ideations in our sample (e.g. [5]).

Widowing (OR = 1.35, CI 95% = 1.12 to 1.63; *P = *.002) was also identified as a variable significantly associated with death ideations in our multivariate analysis, suggesting that, even while controlling for depression, widowing was a specific risk factor for higher levels of depression leading to the wish to be dead.

Finally, having a long-term illness (OR = 1.28, CI 95% = 1.07 to 1.53; *P = *.08) was the last association with death ideations which was significant in the multivariate model, in coherence with previous researches (e.g. [35]).

These results confirm that, besides the high observed effect of depression, some variables remain significantly associated with the presence of death ideations in elders. From these risk factors, widowing (OR = 1.35) and being retired from less than 5 years (OR = 1.46) constitute variables related to social inclusion, on which one can expect a preventive intervention to be targeted at. For instance, Dubé et al. [36] demonstrated the efficacy of a goal management program on level of hope, and abilities to attain personal objectives and achieve projects, these variables showing a moderating effect on lower levels of depression, in a case-control study in Canada targeting 50 to 65 year-old participants who recently retired.

Concerning the social and interpersonal data, none of the three variables (material support received, material support given, community participation) were significantly associated to death ideations in the multivariate model, suggesting that they might not constitute a protective factor against death ideation while controlling for other factors including depression. Thus, some bivariate correlations were significant; Hence, we have shown that receiving material support (assistant for administrative procedures, household chores or help with daily activities–washing, dressing, feeding) was positively associated with the expression of death ideations. This result, which is surprising if we refer to the literature on social support in health psychology [37], could be at least partly explained by the link between material help and dependence in the elderly. We thus assume that the links between material support and death ideations could be due to the mediation of the dependence whose links were also demonstrated to relate to suicide [2].

The bivariate analyses also pointed out the negative association between community participation and death ideations, when other variables were kept out of the model. Individuals who participated in at least one community action (beyond simple membership in a group, but in actions such as exercising voluntary activities, providing regular services to people, attending a class, having an associative or labor-union activity, etc.) appeared to be less at risk of having suicidal thoughts.

In line with the literature, these two results, while non-significant when controlling for other variables, shed light that the primary prevention (in people without depressive symptoms) of death ideations in the elderly requires, on the one hand, fighting against psychological isolation by favoring the presence of an emotional support network, given the sole presence of material support being positively associated with death ideations, and, on the other hand, a minimal level of community participation, its effect being found as soon as individuals start participating in one community action state being involved in an action. However, this effect, non-significant in the multivariate analysis suggests that it exists a strong association between depression and social factors. The direction of this association should stimulate the interest of future researches.

Our recent review of the suicide prevention programs for the elderly had underlined the superiority of interventions based on a psychopathological model [1]. Only the study of Oyama and al. [38] used, within the framework of a suicide prevention initiative, the development of non-specific community groups to increase community participation. This paradigm of intervention seemed promising and needs to be repeated to produce new models of prevention of suicidal risk among the elderly. These new models will have to be based on the social and positive dimensions of health in order to free themselves from directly targeting the behavior to avoid (suicide) in order to make a commitment to its determinants from an integrative perspective.

Nevertheless our results lead to the conclusion that, consistently with the literature, while addressing death ideation or suicide prevention, professionals have to consider first the secondary prevention of depressive symptomatology. Primary prevention of depression strategies through social and community inclusion seems a promising field of investigation, but should be part of a global strategy for social and health policies rather than an alternative to psychosocial interventions targeting depressed elders, whose wish to suicide or being dead seem more related to a lack of autonomy (need of material support) or poor health conditions.

As for perspective from this field of research, one should consider to integrate the notion of “active ageing”, which, for the World Health Organization, is “the process consisting of optimizing the possibilities of good health, participation and safety to increase the quality of life during the old age.” [39]. It seems interesting to base these considerations on integrating the notion of participation into the notions of social inclusion and positive health, particularly in connection with the prevention of depression in later life. Indeed, its impact on mental health has also been the subject of numerous publications [40]. This profound paradigmatic evolution will hopefully lead professionals, on one hand, to favor the principle of subsidiarity in communities, that is, to look for the least professionalized means to meet the needs of the elderly (emotional support, in particular), and, on the other hand, to act in order to develop contexts favorable to social and community participation of the elderly.
